# Impact of chemical reaction on Eyring–Powell fluid flow over a thin needle with nonlinear thermal radiation

**DOI:** 10.1038/s41598-023-48400-1

**Published:** 2023-12-04

**Authors:** Muhammad Nadeem, Imran Siddique, Irfan Saif Ud Din, Fuad A. Awwad, Emad A. A. Ismail, Hijaz Ahmad

**Affiliations:** 1 Department of Mathematics, University of Management and Technology, Lahore, 54770 Pakistan; 2 Department of Mathematics, University of Sargodha, Sargodha, 40100 Pakistan; 3 Department of Quantitative Analysis, College of Business Administration, King Saud University, P. O. Box 71115, 11587 Riyadh, Saudi Arabia; 4 Section of Mathematics, International Telematic University Uninettuno, Corso Vittorio Emanuele II, 39, 00186 Rome, Italy; 5 Near East University, Operational Research Center in Healthcare, Near East Boulevard, 99138 Nicosia/Mersin 10, Turkey; 6 Department of Computer Science and Mathematics, Lebanese American University, Beirut, Lebanon; 7 Center for Applied Mathematics and Bioinformatics, Gulf University for Science and Technology, Mishref, Kuwait

**Keywords:** Engineering, Mathematics and computing, Physics

## Abstract

The thin needle is viewed as a revolutionary object since it has a thinner thickness than a boundary layer. As a consequence, scientific and engineering applications for instance electrical equipment, hot wire anemometers and geothermal power generation are significantly impacted by the flow deformed by a thin moving needle. MHD Eyring–Powell fluid flow over a thin needle perceiving heat source, chemical reaction and nonlinear thermal radiation is the subject of the current investigation. In addition, the present study utilizes the Buongiorno model to examine the special effects of the fluid's Brownian and thermophoretic forces. The solution of the dimensionless form of ODEs is produced by applying exact renovations to the given problem, which is determined by the structure of PDEs. The bvp4c algorithm, based on the finite difference approach is utilized to numerically solve such modified ODEs. For validation, the results obtained indicate good agreement when compared to the literature. Finally, a detailed graphical analysis of key parameters is shown and explained while keeping in mind the physical significance of flow parameters. The results show that as magnetic and fluid parameter values improve, the velocity gradient falls. Increasing heat source and radiation parameters optimises heat transfer rate. The augmentation of the Lewis number and chemical reaction accelerates the rate of mass transfer on the surface. Brownian motion and thermophoresis provide enhanced thermal performance for the fluid temperature. Growing the thermophoresis parameter from 0.1 to 0.3 upsurges the Nusselt number by 5.47% and the Sherwood number by 12.26%.

## Introduction

The estimation of flow and mass transport in non-Newtonian fluids has generated considerable attention and debate plays an essential role in the industry, chemical engineering and biological processes. Examples of these sorts of uses include clay coatings, polymer production, and cosmetics. Due to the wide variety of non-Newtonian fluids, there is no one constitutive relationship between shear rate and stress that can account for all of the key rheological behaviour of such flows. Harris^1^ and Bird et al.^2^ have made enormous strides in our comprehension of non-Newtonian fluid models by including a multiplicity of rheological characteristics. The interest in non-Newtonian fluid flows has greatly risen in the last few decades owing to its extensive usage in the chemical process, food industry, building engineering, petroleum production, commercial and power engineering applications. The flow of non-Newtonian liquids around a stretched surface or a circular cylinder has numerous uses. It has attracted a great deal of curiosity to research various non-Newtonian fluid patterns. The Powell–Eyring fluid is an attractive non-Newtonian fluid that, despite being relatively complex, has numerous advantages among researchers^3^. Malik et al.^4^ conducted on convection in an MHD Eyring–Powell nanofluid over a stretching surface. They demonstrated that raising the mixed convection and fluid parameters accelerated the fluid. Hayat et al.^5,6^ explored Powell–Eyring fluid flow along a moving surface under a convective condition and a free stream. Malik et al.^7^ conducted an analytical study of heat transmission of Eyring–Powell liquid over a stretching cylinder with Vogel's and Reynolds forms of changing viscosity. The radiation's impact on Eyring–Powell fluid across an unstable oriented stretched sheet with a heat source/sink was evaluated by Hayat et al.^8^. The unsteady Eyring–Powell flow in a conduit with a porous surface was examined by Zaman et al.^9^. Rosca and Pop^10^ looked at how an Eyring–Powell fluid flows and transfers heat across a shrinking surface. Relevant components of the Eyring–Powell fluid flow have been examined in several studies^11–14^.

Magnetohydrodynamics (MHD) is the learning of the magnetism and behaviour of conducting liquids. It is sometimes known as hydro-magnetism or magnetohydrodynamics. Magnetic fluids include liquid metal, plasma, electrolytes and salt water. The basic theory underlying MHD is that a magnetic field creates an electric current, which generates Lorentz force, which influences the flowing liquid. The optimal pre-use factor is required for both controlling the cooling rate and achieving acceptable product quality^15^. Afterwards, Eldabe et al.^16^ provided the MHD-free convective heat and mass transfer of Eyring–Powell fluid via a porous medium. The significance of magnetic flux over a thin needle moving vertically was searched by Salleh et al.^17^. MHD Williamson nanofluid moving thin needle flow was hypothetically directed by Khan et al.^18^. View some other notable research on MHD in^19–22^.

Common liquids are often not used extensively in a range of scientific and technical sectors due to their poor heat conductivity. Due to their undeniable thermal impact and unique applications in industries, biological research, and engineering sciences such as nuclear power, paper production, insolation collectors, glass-fibre manufacture, geothermal energy pipe cooling systems and heat transmission of heating nozzles in aircraft equipment, nanoparticles are gaining a lot of attention in the twenty-first century. Nanoparticles are small metallic particles (1–100 nm) having enhanced thermo-physical properties. Nanoparticles are sensitive to many interrelationships that may aid in the formation of particular turbidity patterns or the delimitation of density as well as the generation of nanoparticles and buoyant forces. Recently it was determined that nanofluids have higher thermal conductivity ratings than regular fluids. Choi^23^ established the existence of such a concept by providing experimental validation of nanofluids. Dhanai et al.^24^ utilised the shooting methodology to present a solution of MHD power-law nanofluids caused by a shrinking/stretching surface. The numerical analysis of the MHD micropolar fluid flow on a contracting surface was done by Lund et al.^25^. The mixed convection of nanofluid contained in a thin needle was analyzed by Soid et al.^26^. A thin needle-induced variable fluid characteristic MHD flow of ceramic nanofluid was explored by Nayak et al.^27^. Alsenafi et al.^28^ discussed the heat transfer analysis with Blood-${\text{Fe}}_{{3}} {\text{O}}_{{4}}$ over a thin needle. Scientists worldwide have been fascinated by the dominant qualities of nanofluid, and as a result, amazing investigations have been documented over the past two decades^29–33^.

Thin needle geometry elaborates the blurring surface generated by spinning a parabola about its axis. In such geometries, physical events take place near the disparaging cylindrical tube with quasi-stiffness. The practical value of this particular geometry in a variety of areas, like blood flow challenges, cancer treatment, metal spinning, and led to its acceptance. Many researchers have investigated heat transmission and flow through the use of a moving thin needle. The boundary layer (BL) flow across a narrow needle was invented by Lee^34^. Ishak et al.^35^ accomplished the double solution on a narrow needle. Waini et al.^36^ looked at the radiative flow rate of a fluid flowing on a thin needle. Afridi et al.^37^ exploited heat dissipation to induce entropy for nanofluid flow upon this thin needle. Trimbitas et al.^38^ examined the transmission of heat by convection on a vertical needle theoretically. The reader can further study thermal flows with fluid flowing over a thin needle in Refs.^39–45^.

There are many applications and importance of chemical processes in engineering, geophysics, and industry. Several chemical reactions are carried out in a reactor during industrial chemical operations to renovate less expensive crude resources into higher-standard products. The design of chemical processing machinery, blood pumping, food processing, blood pumping, glass production and other industrial processes all depend heavily on homogeneous/heterogeneous chemical reactions. Due to its benefit, distinct researchers have conducted evaluations for the thermal and mass flux that take into account how chemical reactions influence the flow system. Matin and Pop^46^ used chemical reactive phenomena to describe the convective heat flow for nanofluid over the permeable surface. Mabood et al.^47^ used chemically reactive effects over a needle. Ramzan et al.^48^ examined the stimulus of chemical process upon flow system on fluid flow across a needle. The MHD flowing for nanoparticles upon a horizontal surface has only been described by Makind and Animasaun^49^. Khan et al.^50^ have drawn a connection between the outcomes of a chemical reaction and a Casson fluid moving across a surface. The reader can further study about heat and mass transfer using chemical processes in Refs.^51–56^.

To the extent that we are aware, no authors have taken into account this study. Until now, no research has been directed to demonstrate the 2-dimensional flow of an MHD Eyring–Powell fluid over a thin-needle in the dignity of a chemical reaction. Brownian motion, heat sink/source, thermophoresis and nonlinear thermal radiation are introduced in the report. The interaction of mass, momentum and energy culminates in the entire formulation of a nonlinear mathematical problem. The built-in finite difference method was used to perform nonlinear analysis on the velocity, Sherwood numbers, surface drag force, temperature, local Nusselt and nanoparticle concentration profiles. The authors must respond to the following research queries by the study's conclusion:

- What is the Eyring–Powell fluid's behaviour in the occurrence of a magnetic field parameter, and which fluid is most significantly impacted by the magnetic parameter?
- What is the behaviour of the Eyring–Powell fluids in the bearing of chemical reaction, and which fluid is most severely impacted by a chemical reaction?
- How do the heat sink/source, Brownian motion, and thermophoresis and for which fluid have a dominant impact?
- How does the nonlinear thermal radiation affect the Eyring–Powell fluids?

## Formulation

**Figure 1:**
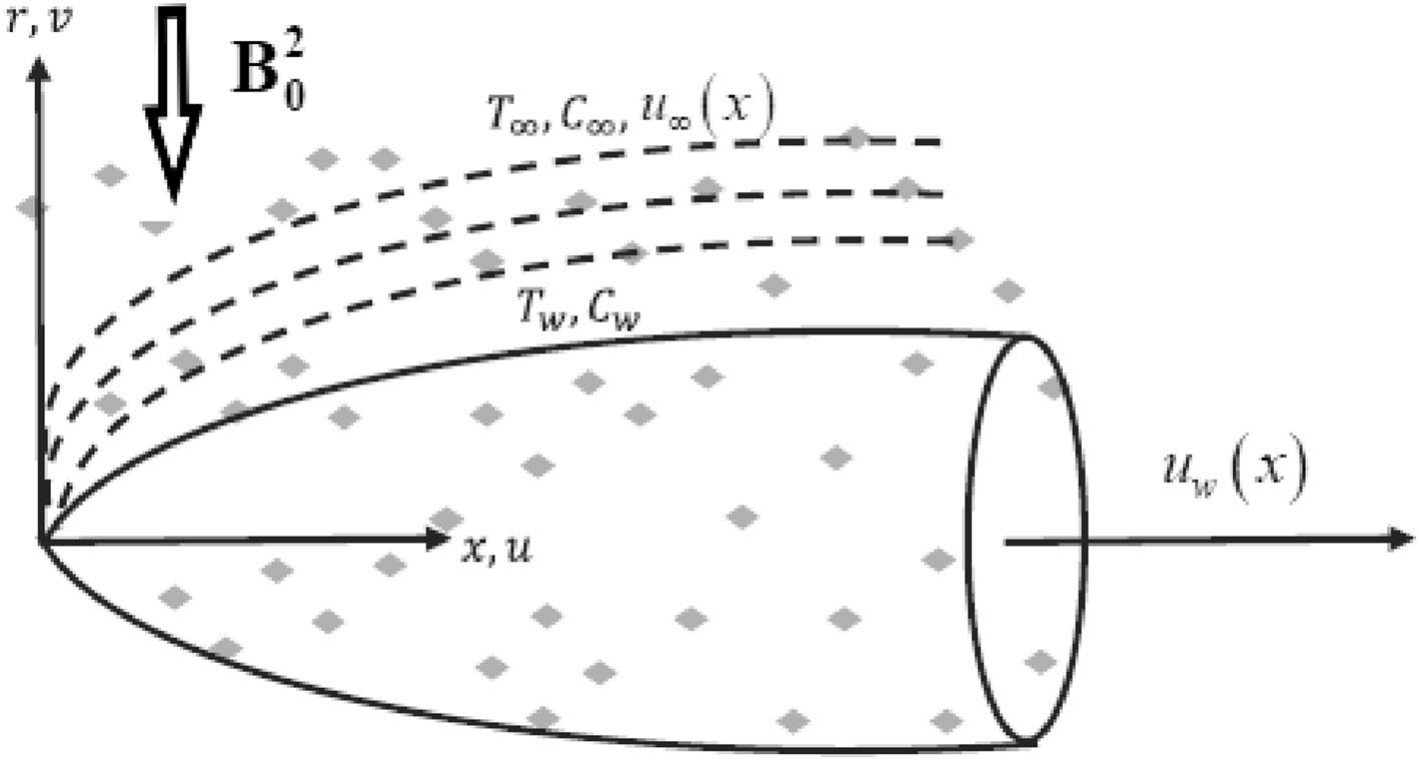
Schematic representation.

Consider the laminar, 2D, steady, MHD, and chemically reactive flow of Eyring–Powell nanofluid over the moving thin-needle in the availability of heat sink/source, thermophoresis, Brownian motion, and nonlinear thermal radiation. As presented in Fig. 1, the needle radius is $r = R = \sqrt {\chi x\nu_{f} /U} ,$ where $r$ is the radial coordinate, $\nu_{f}$ is the kinematic viscosity, $\chi$ is the size or shape, $U = u_{w} + u_{\infty }$ is the composite velocity, $u_{w}$ denoted as the needle is moving horizontally and *x* is the axial coordinate. The heat and momentum BL are thicker than the thin needle, which is thinner yet. The magnetic strength ${\mathbf{\rm B}}_{{\mathbf{0}}}$ is imposed in the radial direction, and there is no pressure gradient on the surface. Furthermore, $T_{w}$ (*K*) and $C_{w}$ (mol/m^3^) are the corresponding amounts at the needle's surface, whereas $T_{\infty }$ and $C_{\infty }$ are considered to be the temperature and concentration at the free stream. Note that the fluid at the surface is thought to contain a concentration and temperature that are higher than the ambient level.

The BL equations for continuity, thermal energy, momentum, and concentration can be stated in cylindrical coordinates under the aforementioned assumptions^7,37,38,41,42^.

1 $$ \frac{\partial }{\partial r}\left( {rv} \right) + \frac{\partial }{\partial x}\left( {ru} \right) = 0, $$

2 $$ \left( {v\frac{\partial u}{{\partial r}} + u\frac{\partial u}{{\partial x}}} \right) = \left( {\frac{{\mu_{f} }}{{\rho_{f} }} + \frac{1}{{\rho_{f} \beta c}}} \right)\left( {\frac{{\partial^{2} u}}{{\partial r^{2} }} + \frac{1}{r}\frac{\partial u}{{\partial r}}} \right) - \frac{1}{{6\rho_{f} \beta c^{3} }}\left( {\frac{1}{r}\left( {\frac{\partial u}{{\partial r}}} \right)^{3} + 3\left( {\frac{\partial u}{{\partial r}}} \right)^{2} \frac{{\partial^{2} u}}{{\partial r^{2} }}} \right) - \frac{{\delta_{f} }}{{\rho_{f} }}B_{o}^{2} u, $$

3 $$ \begin{aligned} \left( {\rho Cp} \right)_{f} \left( {v\frac{\partial T}{{\partial r}} + u\frac{\partial T}{{\partial x}}} \right) & = k_{f} \left( {\frac{{\partial^{2} T}}{{\partial r^{2} }} + \frac{1}{r}\frac{\partial T}{{\partial r}}} \right) + \left( {\rho Cp} \right)_{p} \left( {\frac{{D_{T} }}{{T_{\infty } \Delta C}}\left( {\frac{\partial T}{{\partial r}}} \right)^{2} + D_{B} \frac{\partial T}{{\partial r}}\frac{\partial C}{{\partial r}}} \right) \\ & \quad + Q_{o} \left( {T_{w} - T_{\infty } } \right) - \frac{1}{r}\frac{\partial }{\partial r}\left( {rq_{r} } \right), \\ \end{aligned} $$

4 $$ \left( {v\frac{\partial C}{{\partial r}} + u\frac{\partial C}{{\partial x}}} \right) = \frac{{D_{B} }}{r}\frac{\partial }{\partial r}\left( {r\frac{\partial C}{{\partial r}}} \right) + \frac{{\Delta CD_{T} }}{{T_{\infty } }}\frac{1}{r}\frac{\partial }{\partial r}\left( {\frac{\partial T}{{\partial r}}r} \right) - k_{r}^{*} \left( {C - C_{\infty } } \right), $$

with boundary conditions (BCs):

5 $$ \left. {\begin{array}{*{20}l} {v = 0,\,\,u - u_{w} = 0,\,\,\,\,C - C_{w} = 0,\,\,T - T_{w} = 0,\,\,{\text{at}}\,\,\,\,r = R\left( x \right),} \hfill \\ {T \to 0,\,\,\,\,\,\,C \to 0,\,\,\,u \to u_{\infty } ,\,\,\,\,\,\,\,\,\,\,\,\,{\text{as}}\,\,\,\,r \to \infty .} \hfill \\ \end{array} } \right\} $$

In Eqs. (2)–(4) the notations of viscosity $\left( {\mu_{f} } \right),$ electrical conductivity $\left( {\delta_{f} } \right),$ Heat capacity $\left( {\left( {\rho Cp} \right)_{f} } \right),$ density $\left( {\rho_{f} } \right),$ and thermal conductivity $\left( {k_{f} } \right).$

Now presenting the similarity transformations^41,42^;

6 $$ \eta = \frac{{Ur^{2} }}{{\nu_{f} x}},\quad \psi = \nu_{f} xf\left( \eta \right)\,{\text{and}}\,\theta \left( \eta \right) = \frac{{T - T_{\infty } }}{{T_{w} - T_{\infty } }}, $$

here, the stream function $\left( \psi \right)$ fulfils the continuity-equation with $v = - \frac{1}{r}\frac{\partial \psi }{{\partial x}}\,\,\,{\text{and}}\,\,\,u = \frac{1}{r}\frac{\partial \psi }{{\partial r}}\,$ and governing PDEs are reduced in ODEs using Eq. (6):

7 $$ \left( {1 + \alpha } \right)\left( {2\eta f^{\prime \prime \prime } + 2f^{\prime \prime } } \right) + ff^{\prime \prime } - Mf^{\prime } - \frac{4\alpha \lambda \eta }{3}\left( {\left( {f^{\prime } } \right)^{2} f^{\prime \prime } + \left( {f^{\prime \prime } } \right)^{3} + 12\eta \left( {f^{\prime } } \right)^{2} f^{\prime \prime \prime } } \right) = 0, $$

8 $$ \begin{aligned} & \left( {2\eta \theta^{\prime \prime } + 2\theta^{\prime } } \right) + Nr\left( {\eta \theta^{\prime \prime } + \frac{{\theta^{\prime } }}{2}} \right)\left( {1 + \theta \left( {\theta_{w} - 1} \right)} \right)^{3} + 3\eta Nr\left( {\theta^{\prime } } \right)^{2} \left( {\theta_{w} - 1} \right)\left( {\left( {\theta \left( {\theta_{w} - 1} \right) + 1} \right)^{2} } \right) + \Pr H\theta \\ & \quad + 2\eta \left( {Nt\left( {\theta^{\prime } } \right)^{2} + Nb\theta^{\prime } \phi^{\prime } } \right) = 0, \\ \end{aligned} $$

9 $$ \left( {2\eta \phi^{\prime \prime } + 2\phi^{\prime } } \right) + Le\phi^{\prime } f + \frac{Nt}{{Nb}}\left( {\eta \theta^{\prime \prime } + \theta^{\prime } } \right) - \frac{1}{2}LeK\phi = 0, $$

with BCs becomes

10 $$ \left. {\begin{array}{*{20}l} {f\left( \chi \right) = \frac{\chi a}{2},\,\,\,f^{\prime } \left( \chi \right) = \frac{a}{2},\,\,\,\,\theta \left( \chi \right) = 1,\,\,\,\phi \left( \chi \right) = 1,} \hfill \\ {f^{\prime } \left( \infty \right) = \frac{1 - a}{2},\,\,\,\theta \left( \infty \right) = 0,\,\,\phi \left( \infty \right) = 0,\,\,\,\,\,\eta \to \infty .} \hfill \\ \end{array} } \right\} $$

Furthermore, assume $\chi = \eta$ to represent the needle size.

$$\alpha = \frac{1}{{\beta c\mu_{f} }}$$ the eyring powell first parameter, $$\lambda = \frac{{2U^{3} }}{{c^{2} \nu_{f} }}$$ the eyring powell second parameter, $$\theta_{r} = \frac{{T_{w} }}{{T_{\infty } }}$$ the temperature ratio parameter, $$M = \frac{{\delta_{f} B_{o}^{2} }}{{2\rho_{f} U}}$$ the magnetic parameter, $$a = {{u_{w} } \mathord{\left/ {\vphantom {{u_{w} } U}} \right. \kern-0pt} U}$$ the velocity ratio parameter, $$H = \frac{{Q_{o} }}{{2U\left( {\rho cp} \right)_{f} }}$$ the heat generation parameter, $$Le = \frac{{\nu_{f} }}{{D_{B} }}$$ lewis number, $$Kr = \frac{{Kr^{*} }}{U}$$ chemical reaction parameter, $$Nt = \frac{{\left( {T_{w} - T_{\infty } } \right)\tau D_{T} }}{{T_{\infty } \nu_{f} }}$$ the thermophoresis parameter, $$N_{r} = \frac{{16T_{\infty }^{3} \delta^{**} }}{{k^{**} k_{f} }}$$ the thermal radiation, $${\text{Re}} = \frac{Ux}{{\nu_{f} }}$$ the local Reynolds number, $$Nb = \frac{{\tau D_{B} \left( {C_{w} - C_{\infty } } \right)}}{{\nu_{f} \gamma C}}$$ Brownian motion parameter and $$\Pr = \frac{{\alpha_{f} }}{{k_{f} }}$$ the Prandtl-number.

### Quantities of physical interest

The rate of heat transmission and surface drag force are acknowledged as

11 $$ C_{f} = \frac{{\tau_{w} x}}{{\rho_{f} U^{2} }},\quad Nu_{x} = \frac{{xq_{w} }}{{k_{f} \left( {T_{w} - T_{\infty } } \right)}},\quad {\text{and}}\quad Sh_{x} = \frac{{ - xm_{w} }}{{\left( {C_{w} - C_{\infty } } \right)}}, $$

where $$\tau_{w} = \frac{\partial u}{{\partial r}}\left( {\mu_{f} + \frac{1}{\beta c} - \frac{1}{{6\beta c^{3} }}\left( {\frac{\partial u}{{\partial r}}} \right)^{2} } \right)_{r = \chi }$$, $$q_{w} = \frac{\partial T}{{\partial r}}\left( { - k_{hnf} - \frac{{16\delta^{**} T^{3} }}{{3k^{**} }}} \right)_{r = \chi }$$ and $$m_{w} = \left( {\frac{\partial C}{{\partial r}}} \right)_{r = \chi }$$ are denoted correspondingly as the shear force, thermal, and mass flux.

Exploiting (6), we get

12 $$ \begin{aligned} & \left( {\text{Re}} \right)^{0.5} C_{f} = 4\sqrt \chi \left( {1 + \alpha - \alpha \lambda \left( {f^{\prime \prime } \left( \chi \right)} \right)^{2} } \right)f^{\prime \prime } \left( \chi \right), \\ & \left( {\text{Re}} \right)^{ - 0.5} Nu_{x} = - 2\sqrt \chi \theta^{\prime } \left( \chi \right)\left[ {1 + Nr\left( {1 + \theta \left( \chi \right)\left( {\theta_{w} - 1} \right)} \right)^{3} } \right], \\ & \left( {\text{Re}} \right)^{ - 0.5} Sh_{x} = - 2\sqrt \chi \phi^{\prime } \left( \chi \right). \\ \end{aligned} $$

### Solution procedure

The bvp4c technique is used to show the numerical solution of the altered Eqs. (7) to (9) with BCs (10). As the resulting problem has a two-point boundary value and is highly nonlinear, we must first convert it to first order.

Let's take $$f = y_{1} ,\,\,f^{\prime} = y_{2} ,\,\,f^{\prime\prime} = y_{3} ,\,\,\theta = y_{4} ,\,\,\theta^{\prime} = y_{5} ,\,\,\phi = y_{6} ,\,\,and\,\,\phi^{\prime} = y_{7} ,\,\,$$

13 $$ y_{3}^{\prime } = \left( {\frac{ - 1}{{\left( {1 + \alpha } \right)2x - 16\alpha \lambda x^{2} \left( {y_{2} } \right)^{2} }}} \right)\left[ {\left( {1 + \alpha } \right)2y_{3} + y_{1} y_{3} - My_{2} - \frac{4\alpha \lambda x}{3}\left( {\left( {y_{2} } \right)^{2} y_{3} + \left( {y_{3} } \right)^{3} } \right)} \right], $$

14 $$ y_{5}^{\prime } = \frac{ - 1}{{2x + Nrx\left( {1 + y_{4} \left( {\theta_{w} - 1} \right)} \right)^{3} }}\left[ \begin{gathered} + 3xNr\left( {y_{5} } \right)^{2} \left( {\theta_{w} - 1} \right)\left( {\left( {y_{4} \left( {\theta_{w} - 1} \right) + 1} \right)^{2} } \right) \hfill \\ + \Pr Hy_{4} + 2\eta \left( {Nt\left( {y_{5} } \right)^{2} + Nby_{5} y_{7} } \right) + 2y_{5} \hfill \\ Nr\frac{{y_{5} }}{2}\left( {1 + y_{4} \left( {\theta_{w} - 1} \right)} \right)^{3} \hfill \\ \end{gathered} \right], $$

15 $$ y_{7}^{\prime } = \frac{ - 1}{{2x}}\left[ {2y_{7} + Ley_{7} y_{1} + \frac{Nt}{{Nb}}\left( {xy_{5}^{\prime } + y_{5} } \right) - \frac{1}{2}LeK\phi } \right], $$

with boundary condition becomes,

16 $$ \left. {\begin{array}{*{20}l} {y_{1} \left( \chi \right) = \frac{\chi a}{2},\,y_{2} \left( \chi \right) = \frac{a}{2},\,\,\,y_{4} \left( \chi \right) = 1,\,\,y_{6} \left( \chi \right) = 1,} \hfill \\ {y_{2} \left( \infty \right) = \frac{1 - a}{2},\,y_{4} \left( \infty \right) = 0,\,\,y_{6} \left( \infty \right) = 0.} \hfill \\ \end{array} } \right\} $$

In the present scenario, we choose an appropriate finite $\eta_{\infty }$ value in order to asymptotically satisfy the far-field boundary requirements. In the present study, it is considered that $\eta_{\infty }$ should have a definite value of less than 5 in order for the established conditions to be equally satisfied and for numerical solutions to not just alter. During the computation, the CPU time to calculate the values of profiles in the modelled problem is up to 2.15 s, and the convergence criterion is 10 − 6 with the step size $\Delta \eta$ = 0.001^41,42^.

## Results and discussion

The exploration's focus on determining the manipulate of Brownian motion, nonlinear radiation and thermophoresis on the heat and mass transport properties of Eyring–Powell fluid flow via a thin-needle including chemical process. To get the desired results, the MATLAB-built bvp4c approach is used. For choose various values of the parameters such as $$M = 0.2,$$$$\Pr = 15,$$$$\theta_{r} = 1.2,$$$$Nr = 0.5,$$$$H = 0.1,$$$$\alpha = 0.3,$$$$\lambda = 0.4,$$$$a = 0.2,$$$$\chi = 0.2,$$$$Le = 0.3,$$$$Nt = 0.2,$$ and $$Kr = 0.5.$$ The results of Nusselt number, flow rate, drag force, temperature and Sherwood number were established in the form of tables and figures.

The findings for the code validation are remarkably comparable to the earlier research by Hamid^51^, Ishak et al.^35^, Nadeem et al.^42^, and Song et al.^29^ see Table 1.

**Table 1:** Comparative inspection for $- f^{\prime \prime } \left( \chi \right)$ when $M = Nr = 0.$

$\chi$	Ishak et al.^35^	Nadeem et al.^42^	Song et al.^29^	Hamid^51^	Present Results
0.1	1.28880	1.28881	1.28883	1.28880	1.28877
0.01	8.49240	8.49244	8.49129	8.49241	8.48760
0.001	62.16370	62.16372	62.16245	62.16371	62.15919

**Figure 2:**
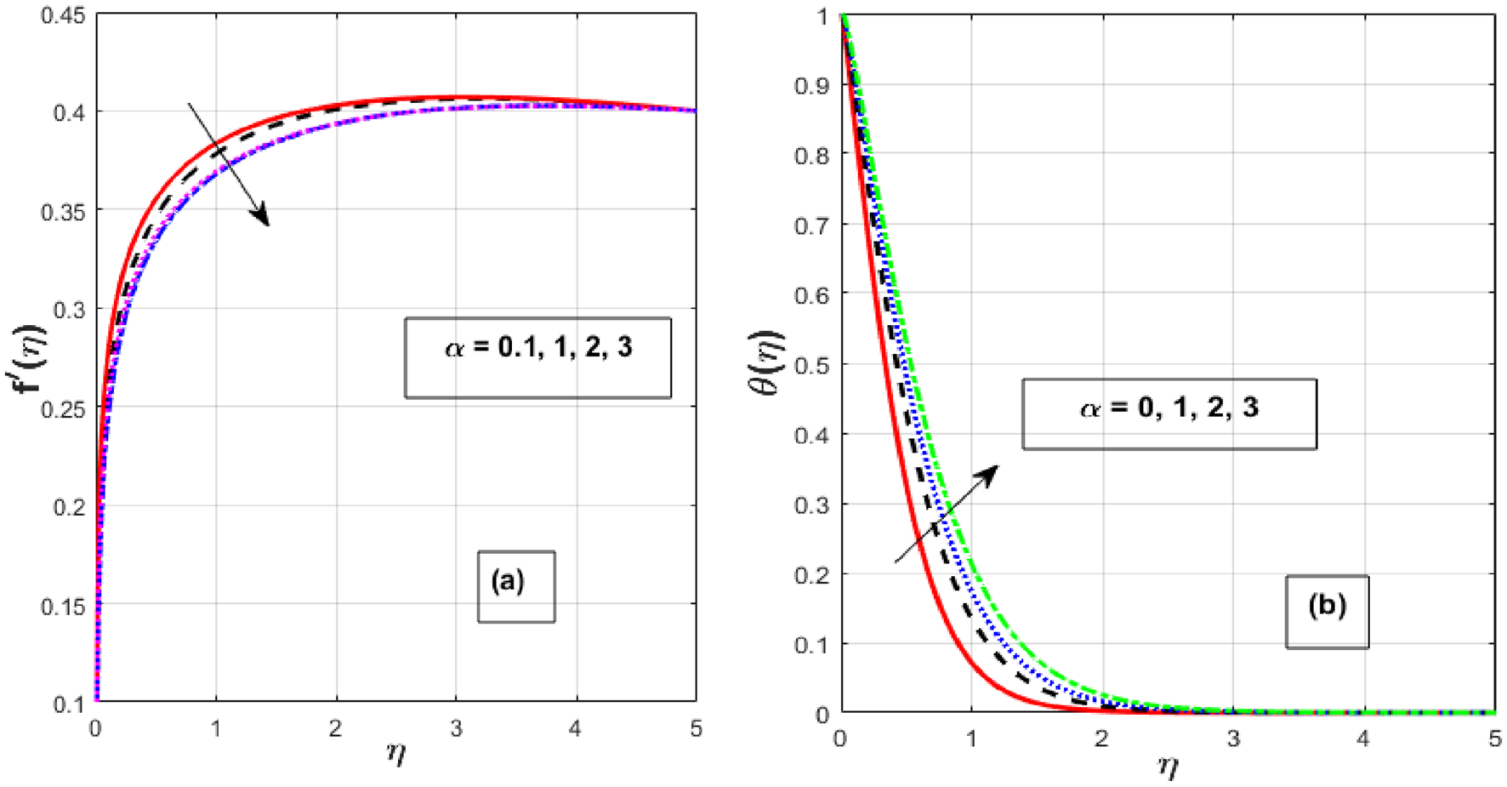
Effect of $\alpha$ on $f^{\prime } \left( \eta \right)$ and $\theta \left( \eta \right).$

**Figure 3:**
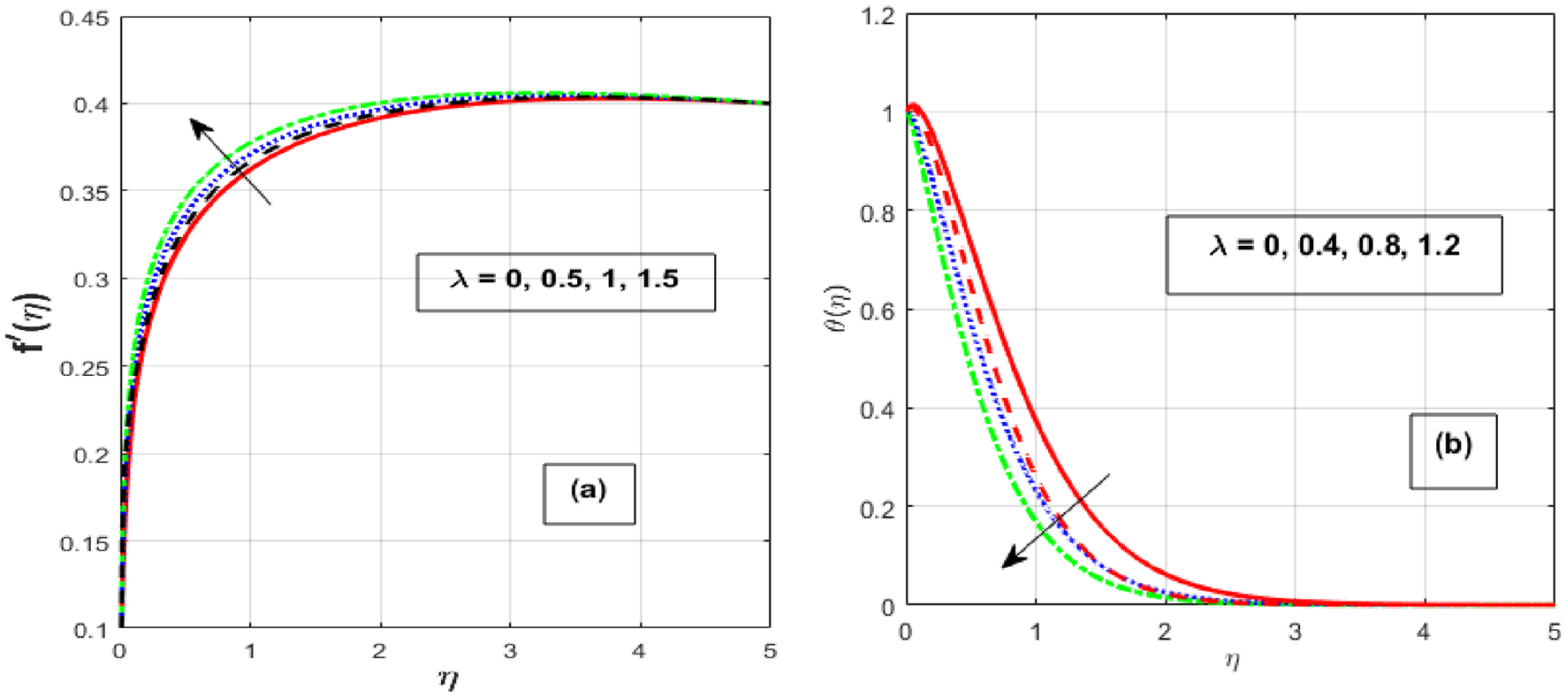
Effect of $\lambda$ on $f^{\prime } \left( \eta \right)$ and $\theta \left( \eta \right).$

**Figure 4:**
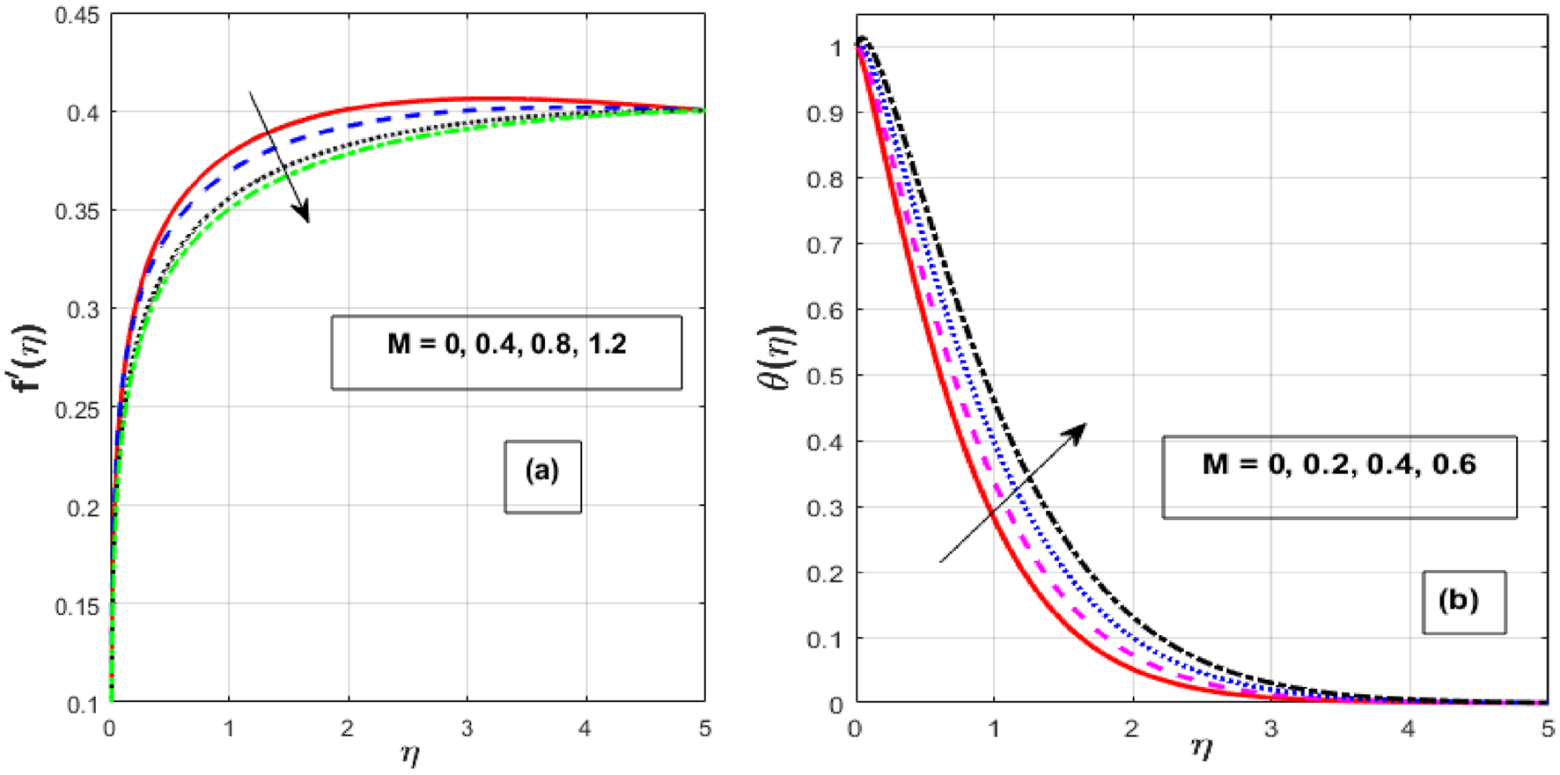
Effect of $M$ on $f^{\prime } \left( \eta \right)$ and $\theta \left( \eta \right).$

**Figure 5:**
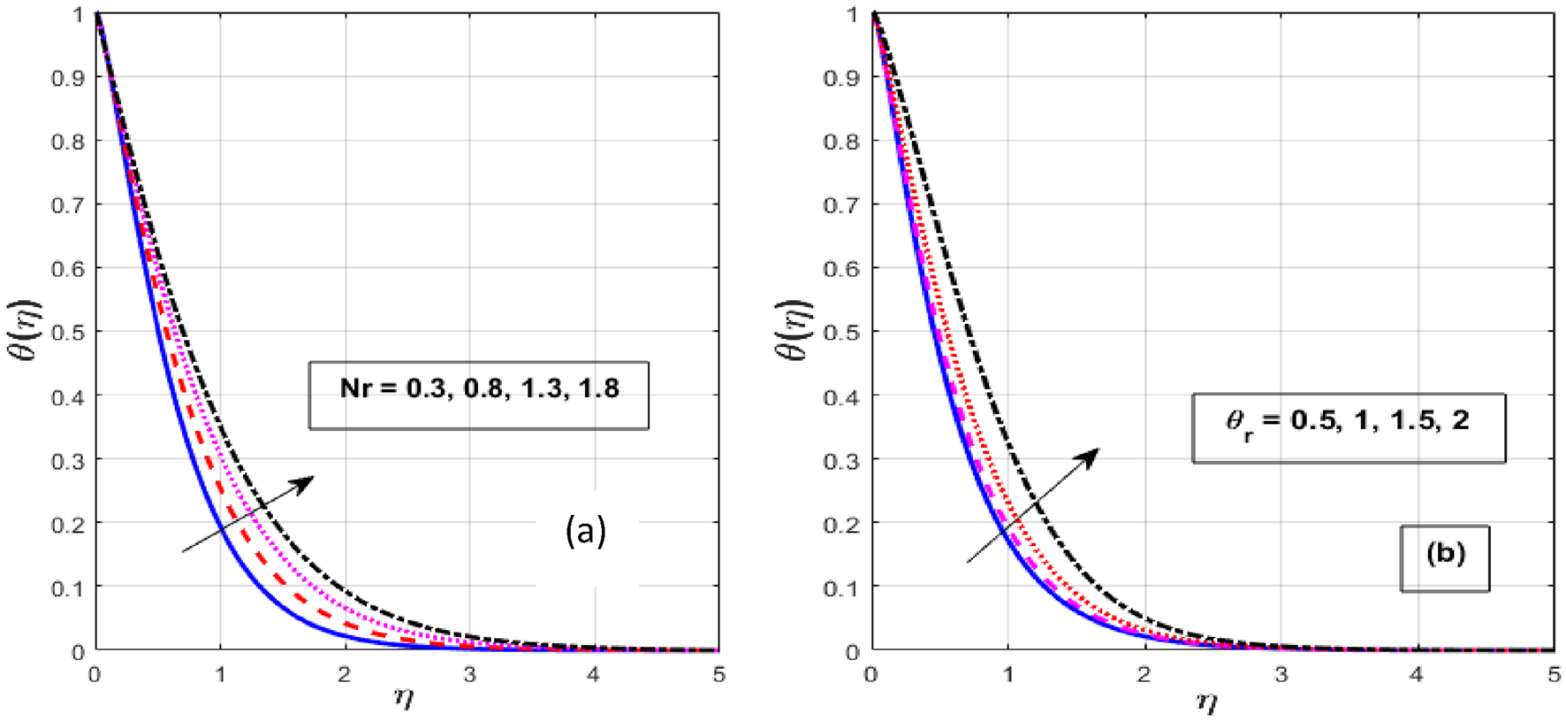
Effect of $Nr$ and $\theta_{r}$ on $\theta \left( \eta \right).$

**Figure 6:**
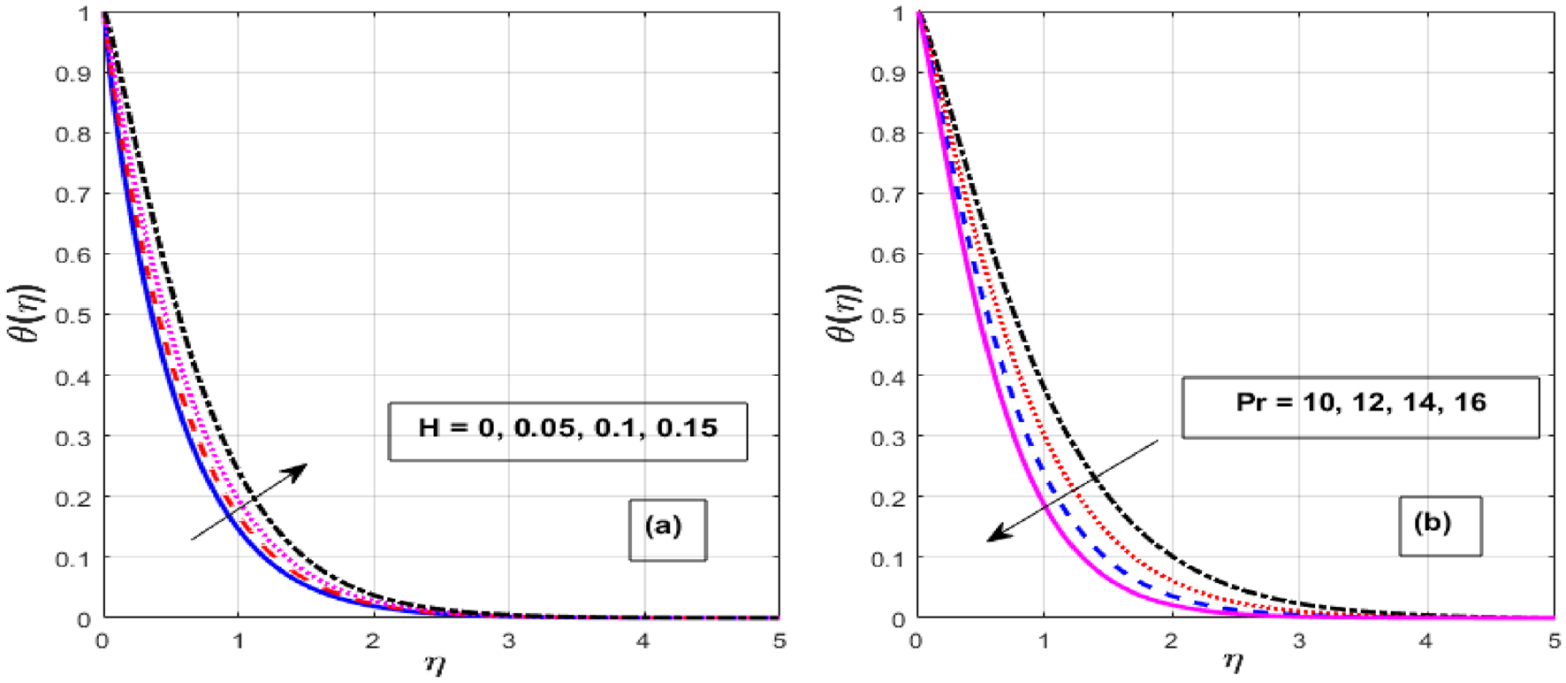
Effect of $H$ and $\Pr$ on $\theta \left( \eta \right).$

Figure 2a,b depicts the consequence of $\alpha$ on the $f^{\prime } \left( \eta \right)$ and $\theta \left( \eta \right).$ The increase in $\alpha$ has resulted in a drop in momentum BL while an increase in heat flux. Physically, increasing the causes it to oppose the free moment of the needle in the flow because resistance forces acting in the opposite direction of the needle movement enhance the surface area of the object. The internal energy and therefore the heat transfer rate develop as a result of these resistive forces. Figure 3a,b depicts the influence of $\lambda$ on the $f^{\prime } \left( \eta \right)$ and $\theta \left( \eta \right).$ As $\lambda$ grow, the velocity profile upsurge whereas fluid temperature decline due to the thermal and momentum BL thickness expands. Eyring–Powell fluid displays shear-thinning properties, therefore their viscosity drops as the shear rate rises. An inverse relationship exists between this characteristic and a non-Newtonian fluid's dynamic viscosity. With an improvement in $\lambda ,$ the flow resistance reduces, and as it does so, the fluid velocity rises. Consequently, it permits the fluid particles to disperse from the surface and reduces the thickness of the thermal BL. The effect of *M* on $f^{\prime } \left( \eta \right)$ and $\theta \left( \eta \right)$ are depicted in Fig. 4a,b. We discovered that increasing the *M* reduces fluid velocity while an increase in fluid temperature. The drag force caused by the produced Lorentz force, which slows down the haphazard motion of fluid particles, is felt by the immersed nanoparticles as the magnetic flux strength steadily increases. Fluid velocity on a needled surface is slowed down by the resistance nanoparticles encounter. The heat released into the system and the thermal boundary profiles exhibit corresponding expansion when the slower fluid increases friction between the fluid layers. Figure 5a,b depicts the impact of the $Nr$ and ${\theta }_{r}$ on the $\theta \left( \eta \right).$ When ${\theta }_{r}$ and $Nr$ are increased, the fluid temperature increases. A larger $\theta_{r}$ indicates that the needle wall and its surrounds are considerably different in temperature. With a temperature rise, the BLs thickness escalates. The radiative component promotes small particle mobility by creating collisions between randomly moving particles, which transforms frictional energy into heat energy. Also, the heat transmission rate to the fluid by radiation increases as the radiation parameter is elevated because it lowers the mean absorption coefficient. The accomplish of the *H* and *Pr* on the $\theta \left( \eta \right)$ are exemplified in Fig. 6a,b. It is well known that as the *H* and *Pr* heighten, so does the heat flow while the temperature and thermal BL thickness drop. The heat-generating mechanism boosts the fluid temperature in the BL zone of the needle by transmitting a substantial amount of heat energy from the needle to the liquid. The influence is more pronounced with lower *Pr* because its heat transmission rate is falling as the BL thickness, because the *Pr* is inversely related to thermal diffusivity.

**Figure 7:**
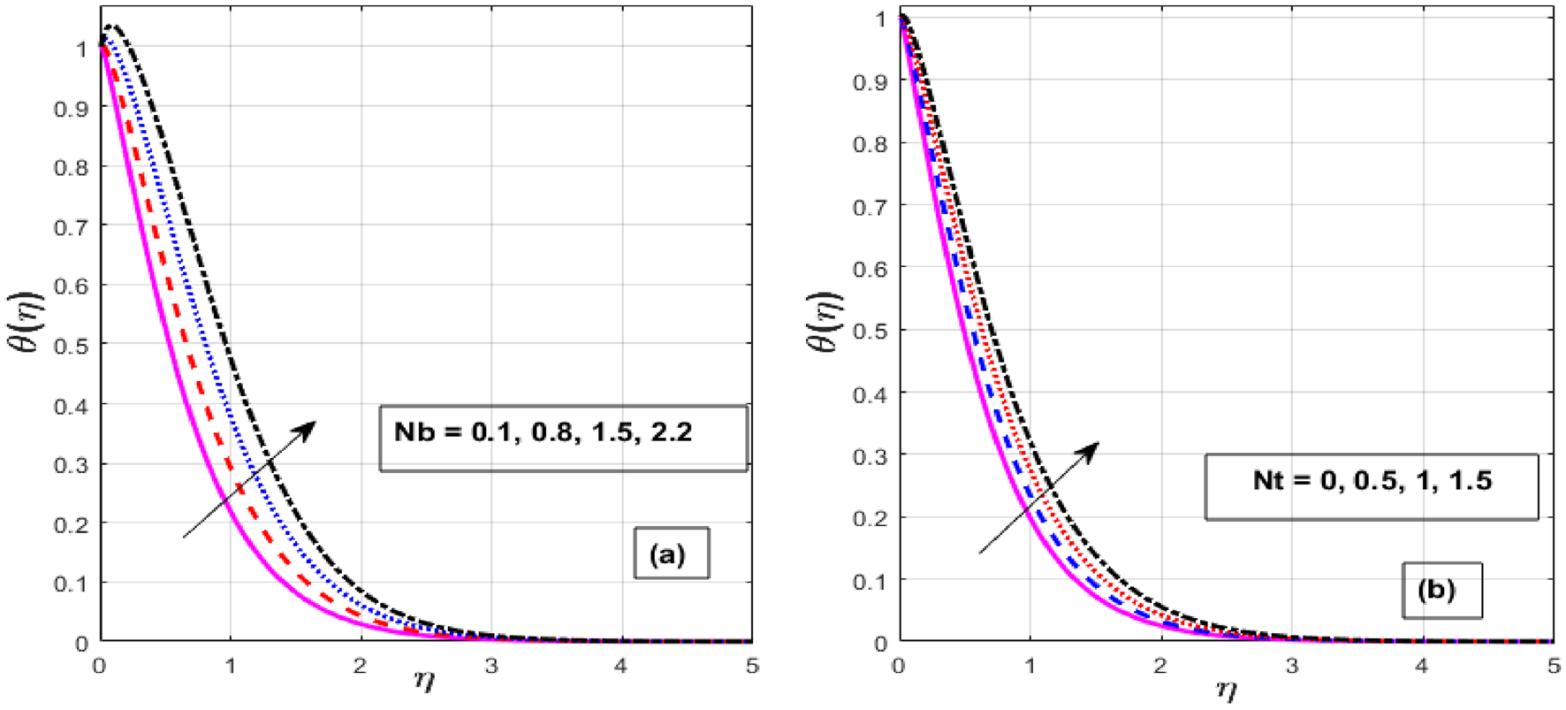
Effect of $Nb$ and $Nt$ on $\theta \left( \eta \right).$

**Figure 8:**
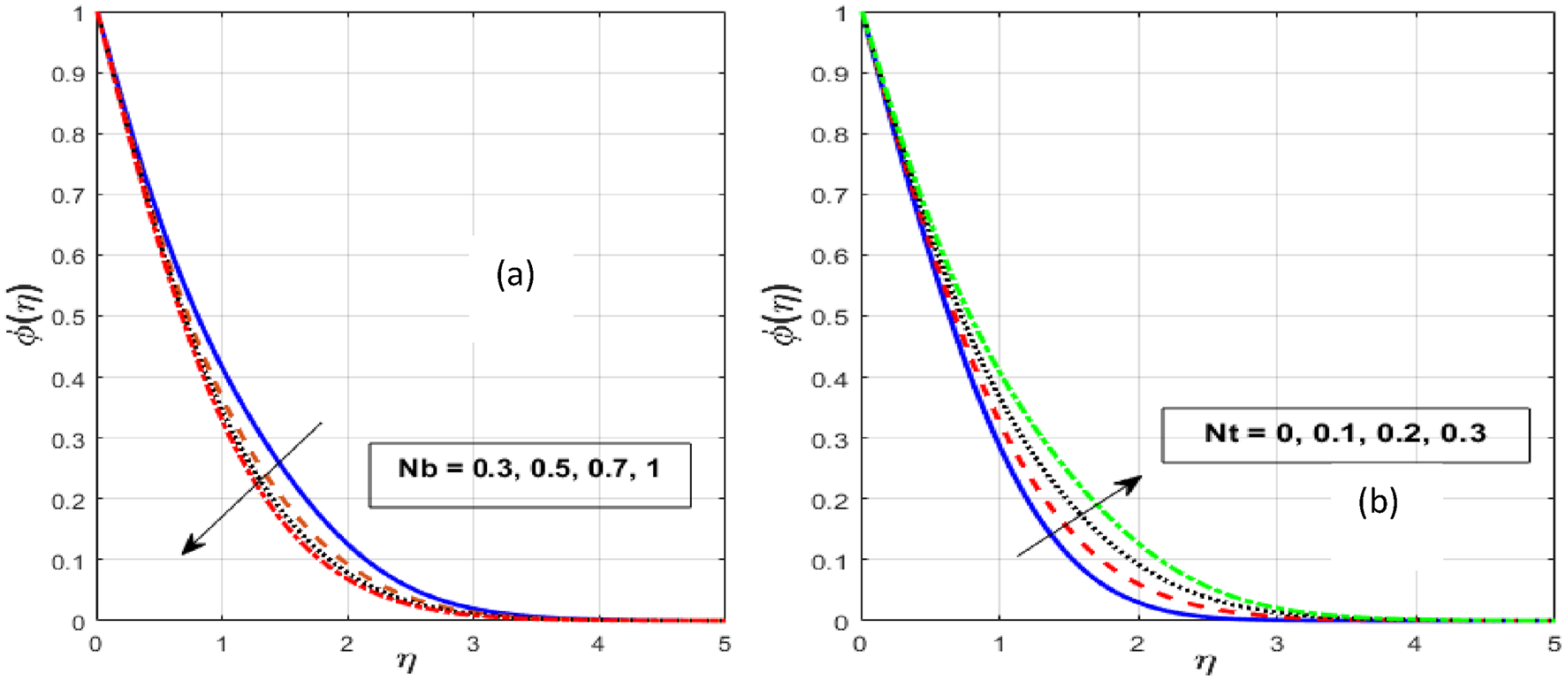
Effect of $Nb$ and $Nt$ on $\phi \left( \eta \right).$

Figure 7a,b depicts the performance of the *Nb* and *Nt* on $\theta \left( \eta \right).$ Strengthening the *Nb* and *Nt*, raising the fluid temperature. The promptly moving molecules or atoms in the base fluid strongly influence the arbitrary moving of nanoparticles spread in it, known as Brownian motion. It occurs when molecules of liquids or gases and nanoparticles interact. Physically, a rise in the *Nb* is accompanied by a noticeable movement of nanoparticles, which raises their kinetic energy and causes them to produce more heat. In addition, this raises the liquid's temperature and the thickness of the thermal BL. This is because particles close to a hot surface produce a force called thermophoresis, which raises the liquid's temperature in the BL area. Thermo-phoresis is a process that pulls tiny particles from a hot surface to a cool one. This concept is supported by the fact that an elevation in *Nt* produces a stronger thermophoretic force, promoting further nanoparticle migrating from a heated surface to a cold ambient liquid, boosting the heat flux and escalating the thermal BL thickness. Figure 8a,b also depicts the effect of *Nt* and *Nb* on $\phi \left( \eta \right).$ The *Nt* and *Nb* have a reverse influence on $\phi \left( \eta \right),$ which means that Nt strengthens the nanoparticle concentration while *Nb* drops it. The randomized mobility and the collision of the macroscopic particles in the fluid raise are realistically related to greater values of *Nb*. Also, Brownian motion is seen to be weaker for larger nanoparticles compared to smaller nanoparticles which reduce the fluid's concentration. As solid nanoparticles are thought to be spherical and evenly dispersed in ordinary fluid, the *Nb* in the Buongiorno pattern rises and falls to its size.

**Figure 9:**
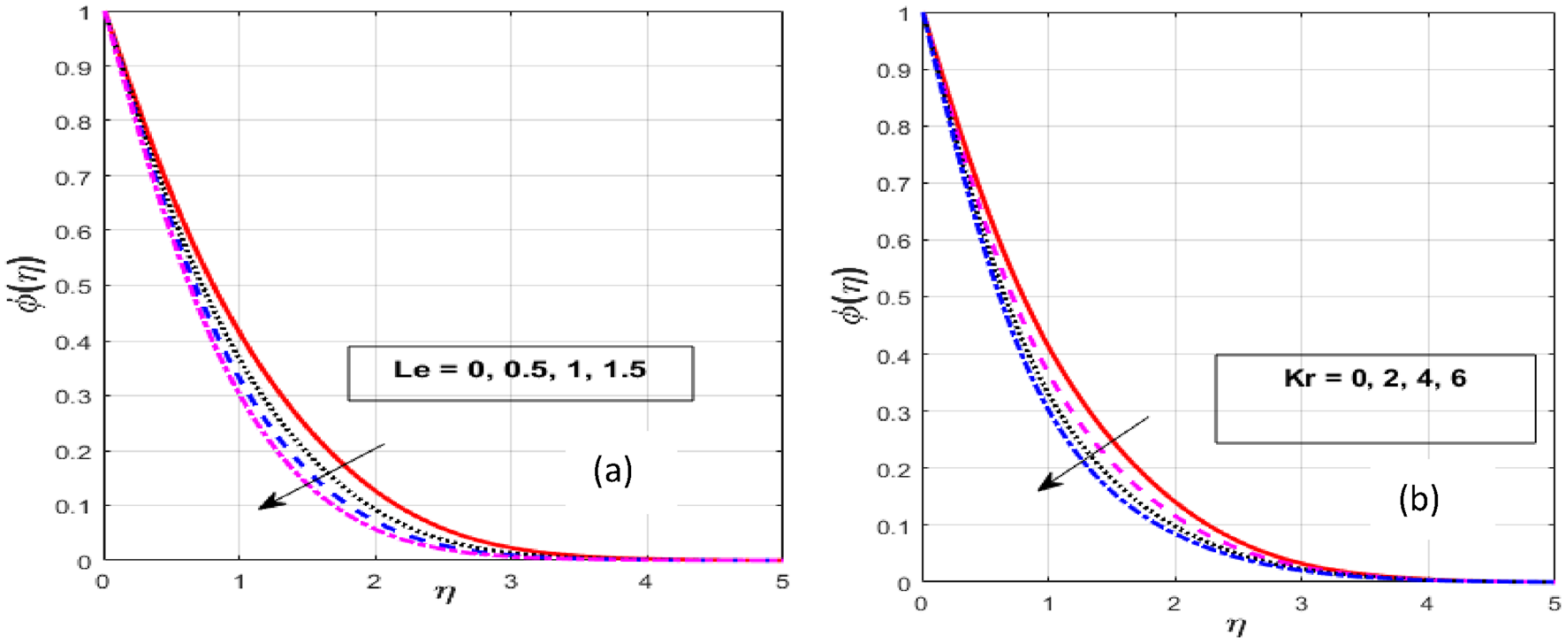
Effect of $Le$ and $Kr$ on $\phi \left( \eta \right).$

Figure 9a,b illustrates that when the *Le* and *Kr* increase, the fluid concentration decrease. Rising *Le* values imply lower molecular movements, which declines the fluid concentration. A fluid with a higher *Le* has a lower Brownian diffusion, causing particles to diffuse deeper into the fluid. Therefore, greater values of the *Le* result in a lower concentration penetration depth. An interaction among two or more molecules that results in the formation of a new compound is known as a chemical reaction. Here, we take into account the constructive kind of chemical reaction, where fluid particles encounter a chemical reaction and merge for higher values of *Kr*. Also, as *Kr* increases, the chemical molecule's diffusivity declines and less diffusion appears. As a consequence, a rise in the *Kr* causes the reactant concentration to drop.

Table 2 displays the impacts of the various constraints on the drag force, heat and mass transfer rate. When the values of alpha and lambda grow, the drag force on the surface increases, but for M, the reverse behaviour occurs. As a consequence, the rapidly rising forces that are inspired by the influences of the strong viscosity are decelerated. This causes the heated fluid motions that are addressing the wall to begin. The heat transfer rate on the surface can be realistically boosted by raising M, lambda, Nt, and Nb, which helps transport more heat into the liquid. The opposite phenomenon has been observed for higher values of alpha, kr, and Le. A faster response rate is indicative of greater values for M, lambda, Nt, kr, and Le. The Sherwood number subsequently rises, however, mass transfer rate exhibits the reverse behaviour for the alpha and Nb parameters.

**Table 2:** Result for $Cf_{x} {\text{Re}}_{x}^{ - 0.5} ,$ $Nu_{x} {\text{Re}}_{x}^{0.5} ,$ and $Sh_{x} {\text{Re}}_{x}^{0.5} .$

*M*	$\alpha$	$\lambda$	*Nt*	*Nb*	*Kr*	*Le*	$Cf_{x} {\text{Re}}_{x}^{ - 0.5}$	$Nu_{x} {\text{Re}}_{x}^{0.5}$	$Sh_{x} {\text{Re}}_{x}^{0.5}$
0.8							1.90932324	1.49636513	0.83242605
0.9							1.09835997	1.56395869	0.84479148
1							0.40909218	1.62438293	0.85671784
	0.2						1.27862446	1.53701022	0.83967500
	0.3						1.90932324	1.49636513	0.83242605
	0.4						2.61881988	1.45899837	0.82605217
		0.2					2.49557941	1.45802547	0.82590922
		0.3					2.55731358	1.45861117	0.82998053
		0.4					2.61881988	1.45958371	0.83242605
			0.1				1.88016488	1.45371897	0.78161808
			0.2				1.88016491	1.49602226	0.83235991
			0.3				1.88016488	1.53785268	0.89078492
				0.3			1.88016491	1.45223387	0.85940818
				0.4			1.88016491	1.49602226	0.83235991
				0.5			1.88016488	1.53649190	0.81608841
					0.4		1.88016491	1.49617616	0.83020956
					0.5		1.88016491	1.49602226	0.83235991
					0.6		1.88016491	1.49587049	0.83448303
						0.2	1.88016491	1.49619945	0.82496549
						0.3	1.88016491	1.49602226	0.83235991
						0.4	1.88016491	1.49581956	0.83935533

## Conclusion

The current work used a binary chemical reaction to investigate a steady 2D MHD Eyring–Powell nanofluid flow over a thin needle that is moving horizontally. The system also includes a heat source/sink, the Begnano model, and nonlinear radiation. The similarity transformation and the bvp4c technique were operated to explain this system of equations, which resulted in the translation of the controlling PDEs into a pair of ODEs. Graphs have been used to discuss and show how different physical parameters affect the flow and thermal profiles. The following results of the study are highlighted:

- When *M* is improved, the occurrence of a Lorentz force declines velocity while enhancing heat transfer, which also enables the Nusselt number of the fluid.
- The *M* lowers the surface drag force.
- As the *Nb* is increased, the variance in nanoparticle concentration weakens, whereas *Nt* enhances the concentration.
- The Eyring–Powell fluid parameters display the opposite behaviour.
- The fluid behaves thermally better because of the thermophoresis and Brownian motion mechanisms. The consequences are also significant in the heating and cooling processes.
- Larger *Nr* and temperature ratio values provide an optimum temperature gradient, which generates the highest heat transfer rate and causes the thermal profile to ascend.
- As the chemical reaction and Lewis number are enhanced, the mass transfer rate is minimized.
- Increased $\lambda$ diminishes the wall friction and rises the Nusselt number whereas $\alpha$ shows the opposite trend.
- The *Le*, alpha and *Kr* increase the shoowerd number while demonstrating the opposite tendency.
- The effects of varied thermal conductivity and variable viscosity can be added to the aforementioned investigation in the future. In addition, hybrid nanofluid may be used in place of the standard nanofluid in the current work.

## Data Availability

The datasets used and analyzed during the current study available from the corresponding author on reasonable request.

## References

[1] Harris J (1977). Rheology and Non-Newtonian Flow.

[2] Bird RB, Curtis CF, Armstrong RC, Hassager O (1987). Dynamics of Polymetric Liquids.

[3] Powell RE, Erying H (1944). Mechanism for the relaxation theory of viscosity. Nature.

[4] Malik MY, Khan I, Hussain A, Salahuddin T (2015). Mixed convection flow of MHD Eyring–Powell nanofluid over a stretching sheet: A numerical study. AIP Adv..

[5] Hayat T, Naza R, Asghar S, Mesloub S (2012). Soret-Dufour effects on three-dimensional flow of third grade fluid. Nucl. Eng. Des..

[6] Hayat T, Iqbal Z, Qasim M, Obaidat S (2012). Steady flow of an Eyring Powell fluid over a moving surface with convective boundary conditions. Int. J. Heat Mass Transf..

[7] Malik MY, Hussain A, Nadeem S (2013). Boundary layer flow of an Eyring–Powell model fluid due to a stretching cylinder with variable viscosity. Scientia Iranica Trans. B Mech. Eng..

[8] Hayat T, Asad S, Mustafa M, Alsaedi A (2014). Radiation effects on the flow of Powell–Eyring fluid past an unsteady inclined stretching sheet with non-uniform heat source/sink. PLoS ONE.

[9] Zaman H, Shah MA, Ibrahim M (2013). Unsteady incompressible Couette flow problem for the Eyring–Powell model with porous walls. Am. J. Comput. Math..

[10] Rosca AV, Pop I (2014). Flow and heat transfer of PowellEyring fluid over a shrinking surface in a parallel free stream. Int. J. Heat Mass Transf..

[11] Li YX, Khan MI, Gowda RP, Ali A, Farooq S, Chu YM, Khan SU (2021). Dynamics of aluminum oxide and copper hybrid nanofluid in nonlinear mixed Marangoni convective flow with entropy generation: Applications to renewable energy. Chin. J. Phys..

[12] Sarada K, Gamaoun F, Abdulrahman A, Paramesh SO, Kumar R, Prasanna GD, Gowda RP (2022). Impact of exponential form of internal heat generation on water-based ternary hybrid nanofluid flow by capitalizing non-Fourier heat flux model. Case Stud. Therm. Eng..

[13] Punith Gowda RJ, Sarris IE, Kumar N, Kumar R, Prasannakumara BC (2022). A three-dimensional non-Newtonian magnetic fluid flow induced due to stretching of the flat surface with chemical reaction. J. Heat Transf..

[14] Siddique I, Nadeem M, Awrejcewicz J, Pawłowski W (2022). Soret and Dufour effects on unsteady MHD second-grade nanofluid flow across an exponentially stretching surface. Sci. Rep..

[15] Gregory TS, Cheng R, Tang G, Mao L, Tse ZTH (2016). The magnetohydrodynamice_ect and its associated material designs for biomedical applications: A state-of-the-art review. Adv. Funct. Mater..

[16] Eldabe NTM, Sallam SN, Abou-zeid MY (2012). Numerical study of viscous dissipation effect on free convection heat and mass transfer of MHD non-Newtonian fluid flow through a porous medium. J. Egypt. Math. Soc..

[17] Salleh SNA, Bachok N, Arifin NM, Ali FM, Pop I (2018). Magnetohydrodynamics flow past a moving vertical thin needle in a nanofluid with stability analysis. Energies.

[18] Khan A, Saeed A, Tassaddiq A, Gul T, Mukhtar S, Kumam P, Ali I, Kumam W (2021). Bio-convective micropolar nanofluid flow over thin moving needle subject to Arrhenius activation energy, viscous dissipation and binary chemical reaction. Case Stud. Therm. Eng..

[19] Sreenivasa BRJ, Faqeeh A, Alsaiari A, Alzahrani HA, Malik MY (2022). Numerical study of heat transfer mechanism in the flow of ferromagnetic hybrid nanofluid over a stretching cylinder. Waves Random Complex Media.

[20] Jamshed W, Gowda RJP, Kumar RN, Prasannakumara BC, Nisar KS, Mahmoud O, Rehman A, Pasha AA (2022). Entropy production simulation of second-grade magnetic nanomaterials flowing across an expanding surface with viscidness dissipative flux. Nanotechnol. Rev..

[21] Siddique I, Nadeem M, Khan I, Jamil RN, Shamseldin MA, Akgül A (2022). Analysis of fuzzified boundary value problems for MHD Couette and Poiseuille flow. Sci. Rep..

[22] Nadeem M, Siddique I, Jarad F, Jamil RN (2021). Numerical study of MHD third-grade fluid flow through an inclined channel with ohmic heating under fuzzy environment. Math. Probl. Eng..

[23] Choi SUS (1995). Enhancing thermal conductivity of fluids with nanoparticles. ASME Publ. Fed..

[24] Dhanai R, Rana P, Kumar L (2015). Multiple solutions of MHD boundary layer flow and heat transfer behavior of nanofluids induced by a power-law stretching/shrinking permeable sheet with viscous dissipation. Powder Technol..

[25] Lund LA, Omar Z, Khan I, Raza J, Sherif ESM, Seikh AH (2020). Magnetohydrodynamic (MHD) flow of micropolar fluid with effects of viscous dissipation and joule heating over an exponential shrinking sheet: Triple solutions and stability analysis. Symmetry.

[26] Soid SK, Ishak A, Pop I (2017). Boundary layer flow past a continuously moving thin needle in a nanofluid. Appl. Therm. Eng..

[27] Nayak MK, Mehmood R, Mishra S, Misra A, Muhammad T (2021). Thermal and velocity slip effects in mixed convection flow of magnetized ceramic nanofluids over a thin needle with variable physical properties. Waves Random Complex Media.

[28] Alsenafi A, Ferdows M (2022). Similarity and finite difference solution on biomagnetic flow and heat transfer of blood-Fe_3_O_4_ through a thin needle. J. Math..

[29] Song YQ, Hamid A, Khan MI, Gowda RP, Kumar RN, Prasannakumara BC, Khan SU, Khan MI, Malik MY (2021). Solar energy aspects of gyrotactic mixed bioconvection flow of nanofluid past a vertical thin moving needle influenced by variable Prandtl number. Chaos Solitons Fractals.

[30] Wang F, Rani SP, Sarada K, Gowda RP, Zahran HY, Mahmoud EE (2022). The effects of nanoparticle aggregation and radiation on the flow of nanofluid between the gap of a disk and cone. Case Stud. Thermal Eng..

[31] Siddique I, Zulqarnain RM, Nadeem M, Jarad F (2021). Numerical simulation of MHD Couette flow of a fuzzy nanofluid through an inclined channel with thermal radiation effect. Comput. Intell. Neurosci..

[32] Bilal M, Siddique I, Borawski A, Raza A, Nadeem M, Sallah M (2022). Williamson magneto nanofluid flow over partially slip and convective cylinder with thermal radiation and variable conductivity. Sci. Rep..

[33] Jyothi AM, Kumar RN, Gowda RP, Prasannakumara BC (2021). Significance of Stefan blowing effect on flow and heat transfer of Casson nanofluid over a moving thin needle. Commun. Theor. Phys..

[34] Lee LL (1967). Boundary layer above a thin needle. Phys. Fluids.

[35] Ishak A, Nazar R, Pop I (2007). Boundary layer flow over a continuously moving thin needle in a parallel free stream. Chin. Phys. Lett..

[36] Waini I, Ishak A, Pop I (2019). Hybrid nanofluid flow and heat transfer past a vertical thin needle with prescribed surface heat flux. Int. J. Numer. Methods Heat Fluid Flow.

[37] Afridi MI, Tlili I, Goodarzi M, Osman M, Khan NA (2019). Irreversibility analysis of hybrid nanofluid flow over a thin needle with effects of energy dissipation. Symmetry.

[38] Trimbitas R, Grosan T, Pop I (2014). Mixed convection boundary layer flow along vertical thin needles in nanofluids. Int. J. Numer. Methods Heat Fluid Flow.

[39] Alsulami MD, Abdulrahman A, Kumar RN, Punith Gowda RJ, Prasannakumara BC (2023). Three-dimensional swirling flow of nanofluid with nanoparticle aggregation kinematics using modified Krieger-Dougherty and Maxwell-Bruggeman models: A finite element solution. Mathematics.

[40] Alsulami MD, Kumar N, Punith-Gowda RJ, Prasannakumara BC (2023). Analysis of heat transfer using Local thermal non-equilibrium conditions for a non-Newtonian fluid flow containing Ti6Al4V and AA7075 nanoparticles in a porous media. ZAMM-J. Appl. Math. Mech./Zeitschrift für Angewandte Mathematik und Mechanik.

[41] Bilal M, Urva Y (2021). Analysis of non-Newtonian fluid flow over fine rotating thin needle for variable viscosity and activation energy. Arch. Appl. Mech..

[42] Nadeem M, Siddique I, Ali R, Riahi MK, Mousa AAA, Khan I, Hafeez HM, Azam M (2022). Dynamics of non-Newtonian tangent hyperbolic liquids conveying tiny particles on objects with variable thickness when lorentz force and thermal radiation are significant. Front. Phys..

[43] Zulqarnain RM, Nadeem M, Siddique I, Ahmad H, Askar S, Samar M (2023). Heat transfer analysis of Maxwell tri-hybridized nanofluid through Riga wedge with fuzzy volume fraction. Sci. Rep..

[44] Nadeem M, Siddique I, Riaz Z, Makhdoum BM, Zulqarnain RM, Sallah M (2023). Numerical study of unsteady tangent hyperbolic fuzzy hybrid nanofluid over an exponentially stretching surface. Sci. Rep..

[45] Nadeem M, Siddique I, Bilal M, Anjum K (2023). Numerical study of MHD Prandtl Eyring fuzzy hybrid nanofluid flow over a wedge. Numer. Heat Transf. Part A Appl..

[46] Matin MH, Pop I (2013). Forced convection heat and mass transfer flow of a nanofluid through a porous channel with a first order chemical reaction on the wall. Int. Commun Heat Mass Transf..

[47] Mabood F, Nayak MK, Chamkha AJ (2019). Heat transfer on the cross flow of micropolar fluids over a thin needle moving in a parallel stream influenced by binary chemical reaction and Arrhenius activation energy. Eur. Phys. J. Plus.

[48] Ramzan M, Shaheen N, Kadry S, Ratha Y, Nam Y (2020). Thermally stratified darcy forchheimer flow on a moving thin needle with homogeneous heterogeneous reactions and non-uniform heat source/sink. Appl. Sci..

[49] Makinde OD, Animasaun IL (2016). Thermophoresis and Brownian motion effects on MHD bioconvection of nanofluid with nonlinear thermal radiation and quartic chemical reaction past an upper horizontal surface of a paraboloid of revolution. J. Mol. Liq..

[50] Khan MI, Waqas M, Hayat T, Alsaedi A (2017). A comparative study of Casson fluid with homogeneous-heterogeneous reactions. J. Colloid Interface Sci..

[51] Hamid A (2020). Terrific effects of Ohmic-viscous dissipation on Casson nanofluid flow over a vertical thin needle: Buoyancy assisting & opposing flow. J. Mater. Res. Technol..

[52] Dawar A, Acharya N (2022). Unsteady mixed convective radiative nanofluid flow in the stagnation point region of a revolving sphere considering the influence of nanoparticles diameter and nanolayer. J. Indian Chem. Soc..

[53] Acharya N, Mabood F, Badruddin IA (2022). Thermal performance of unsteady mixed convective Ag/MgO nanohybrid flow near the stagnation point domain of a spinning sphere. Int. Commun. Heat Mass Transf..

[54] Acharya N, Mabood F, Shahzad SA, Badruddin IA (2022). Hydrothermal variations of radiative nanofluid flow by the influence of nanoparticles diameter and nanolayer. Int. Commun. Heat Mass Transf..

[55] Acharya N (2021). Spectral simulation to investigate the effects of nanoparticle diameter and nanolayer on the ferrofluid flow over a slippery rotating disk in the presence of low oscillating magnetic field. Heat Transf..

[56] Acharya N (2020). Framing the impacts of highly oscillating magnetic field on the ferrofluid flow over a spinning disk considering nanoparticle diameter and solid–liquid interfacial layer. J. Heat Transf..

